# Patient‐specific adaptive planning margin for whole bladder radiation therapy

**DOI:** 10.1002/acm2.14617

**Published:** 2024-12-23

**Authors:** Zhexuan Zhang, Chieh‐Wen Liu, Jeremy D. Donaghue, Eric J. Murray, Omar Mian, Ping Xia

**Affiliations:** ^1^ Department of Radiation Oncology Taussig Cancer Institute Cleveland Clinic Cleveland Ohio USA

**Keywords:** adaptive margin, bladder

## Abstract

**Background:**

Whole bladder irradiation is an organ preservation treatment approach for muscle‐invasive bladder cancer (MIBC). Conventional planning margins, typically 15–20 mm, increase normal tissue toxicity and limit possible dose escalation.

**Purpose:**

The study aimed to develop a patient‐specific adaptive margin recipe for whole bladder irradiation to minimize the planning target volume (PTV) while preserving adequate dose coverage.

**Methods:**

Sixteen patients who received whole‐bladder irradiation were retrospectively selected for this study. We proposed a patient‐specific anisotropic adaptive margin recipe, derived from the first five fractions of kV‐CBCTs, to account for inter‐fractional bladder changes. This recipe was validated using kV‐CBCTs from fractions six to ten and the final five fractions. The goal was to achieve a residual volume, defined as the percentage of daily bladder volume (V_daily_) outside the PTV, of less than 5%. Adaptive and conventional plans were created using proposed and conventional margins, respectively. A dosimetric comparison of targets and organs‐at‐risk (OARs) was performed between the two approaches.

**Results:**

(V_daily_) decreased throughout the treatment course. The most notable inter‐fractional bladder variations were in the superior and anterior directions. The patient‐specific anisotropic adaptive margins, averaging 6 mm (± 2.9 mm), achieved a residual volume of less than 5%. Compared to conventional planning, the adaptive approach reduced PTV volume by an average of 135.3 cc (± 46.6 cc). A significant correlation (*p* < 0.05) was identified between residual volume and adaptive margins in the anterior, superior, left, and right directions. Using the proposed adaptive margins, the median residual volume was 0.71% (interquartile range 0.09%−3.55%), and the median (V_daily_) receiving the prescribed dose was 99.1% (interquartile range 95.3%−99.9%). Adaptive plans demonstrated superior OAR sparing compared to conventional plans.

**Conclusions:**

The proposed patient‐specific adaptive margin recipe for whole bladder irradiation resulted in margins smaller than conventional ones, optimized normal tissue sparing, and maintained adequate PTV coverage.

## 1 INTRODUCTION

To account for the significant inter‐fractional variation in the bladder, whole bladder irradiation for muscle‐invasive bladder cancer (MIBC) requires a large margin, typically 15–20 mm isotropically, for the planning target volume (PTV) from the clinical target volume (CTV).^1^, ^2^ Studies have shown that an empty bladder improves reproducibility throughout the treatment course.^2^, ^3^ Therefore, in order to further reduce the daily bladder variation and planning margin, treatment protocols for whole bladder irradiation are typically treated with an empty bladder.^2^

Traditionally, radiation treatment plans were developed based on the planning CT, which provided a snapshot of the patient's anatomy at the time of CT simulation. The expansion from the CTV to the PTV was based on patient population statistics rather than patient‐specific anatomy. The concept of adaptive radiotherapy was introduced to improve the therapeutic ratio by modifying the radiotherapy plan based on patient‐specific anatomy and physiological function acquired early in the treatment course.^4^ Advancements in daily imaging guidance have enabled us to identify inter‐fractional bladder variations due to differences in bladder filling and inhomogeneous bladder wall elasticity among patients.^5^, ^6^, ^7^, ^8^, ^9^, ^10^, ^11^, ^12^ The adaptive planning approach allows clinicians to create patient‐specific treatment plans tailored to individual anatomical variations, which can further reduce normal tissue toxicity and allow potential dose escalation to target.^13^

There are three major strategies for performing adaptive radiation therapy on bladder cancer patients: offline adaptive planning, plan of the day selection (PoD), and daily online adaptation.^1^, ^14^ Offline adaptation typically repeats the entire process from simulation to planning in the middle of the treatment course, addressing anatomical changes that occur mid‐course.^2^, ^15^ The PoD strategy preemptively develops several radiotherapy plans using different PTV expansions and treats the patient with one of these prepared plans based on the daily kV‐CBCTs^8^, ^14^, ^16^, ^17^, ^18^ Online adaptation uses an integrated imaging, planning, and treatment system to develop an adaptive plan in real‐time, while the patient is on the treatment table.^9^, ^19^, ^20^ All three methods aim to reduce normal tissue toxicity while maintaining target coverage. Although online adaptive planning is appealing, it is resource‐demanding, which is why many centers do not offer this option. Additionally, the duration of the real‐time adaptation workflow can be extended, typically ranging from 13 to 40 min. During the turnaround time, bladder volume may change. To account for uncertainty during this workflow, additional margins (5–15 mm) are applied to the online adaptive reference plan. The offline and PoD options predict bladder change and generate treatment plans in advance, making them implementable in many centers without the need for a specific platform. Furthermore, the duration from patient setup and imaging to treatment delivery is greatly reduced, ensuring minimal bladder volume changes at treatment time compared to the real‐time adaptive process. In this study, we examined volume changes derived from pre‐void and post‐void CT scans acquired during simulation and daily KV‐CBCTs for a cohort of patients. We developed a patient‐specific adaptive planning margin recipe based on bladder volume variation observed in the first five fractions. This recipe was used to generate adaptive plans for the subsequent treatment course after the first five fractions. Further, we conducted a retrospective analysis of the geometric difference between the adaptive PTV and the daily bladder volume (V_daily_). A dosimetric analysis was also performed on the adaptive plans to quantify target coverage and normal tissue sparing.

## 2 METHOD

### 2.1 Patient selection, simulation, and treatment regimen

Sixteen patients who underwent whole bladder irradiation between 2019 and 2021 were retrospectively selected for this study from a clinical database, that was approved by the local Institutional Review Board. Six patients received treatment with 64 Gy in 32 fractions, while the remaining ten were treated with 55 Gy in 20 fractions. Each patient had two CT scans acquired at simulation, one pre‐void and one post‐void. Patients were instructed to consume 250 cc of fluid and refrain from urinating for 1 hour prior to the pre‐void CT acquisition. They were then directed to void their bladder immediately before the acquisition of the post‐void CT. The post‐void CT was used for planning, with the bladder, small bowel, large bowel, rectum, and femoral heads contoured by the attending radiation oncologists. The pre‐void bladder contour was transferred to the post‐void CT using rigid image registration. All patients underwent daily KV‐CBCT using Varian TrueBeam machines.

During the course of the treatment, patients were instructed to void their bladders before entering the treatment room. Due to variations in bladder function and patient age, the bladder changes can vary day‐to‐day and between patients. To evaluate the variations in bladder volume and shape among these patients, we manually contoured the bladder on a total of 240 daily KV‐CBCTs from the first five fractions, fractions six to ten, and the final five fractions of treatment. For each patient, the daily KV‐CBCTs were registered to the post‐void planning CT by aligning to the bladder neck. The daily bladder contours were then transferred to the planning CT for further volume and shape analysis.

### 2.2 Determining patient‐specific adaptive margins

#### 2.2.1 Residual bladder volume and normalized directional distance

To incorporate volume change during the course of treatment into the adaptive margin calculation, we computed the residual bladder volume ∆V (%), defined as the percentage of (V_daily_) exceeding the planning bladder volume (post‐void bladder V_post_). We also implemented an exclusion criterion to filter out occasional outliers when a patient was non‐compliant and needed to void their bladder again before proceeding with the daily treatment. The exclusion criterion was chosen based on the distribution of the ∆V (%) to ensure that individual bladder variation would fall within a predictable range. Lastly, we utilized the Dice Similarity Coefficient (DSC) to quantify the geometric difference between V_daily_ and V_post_.

**FIGURE 1:**
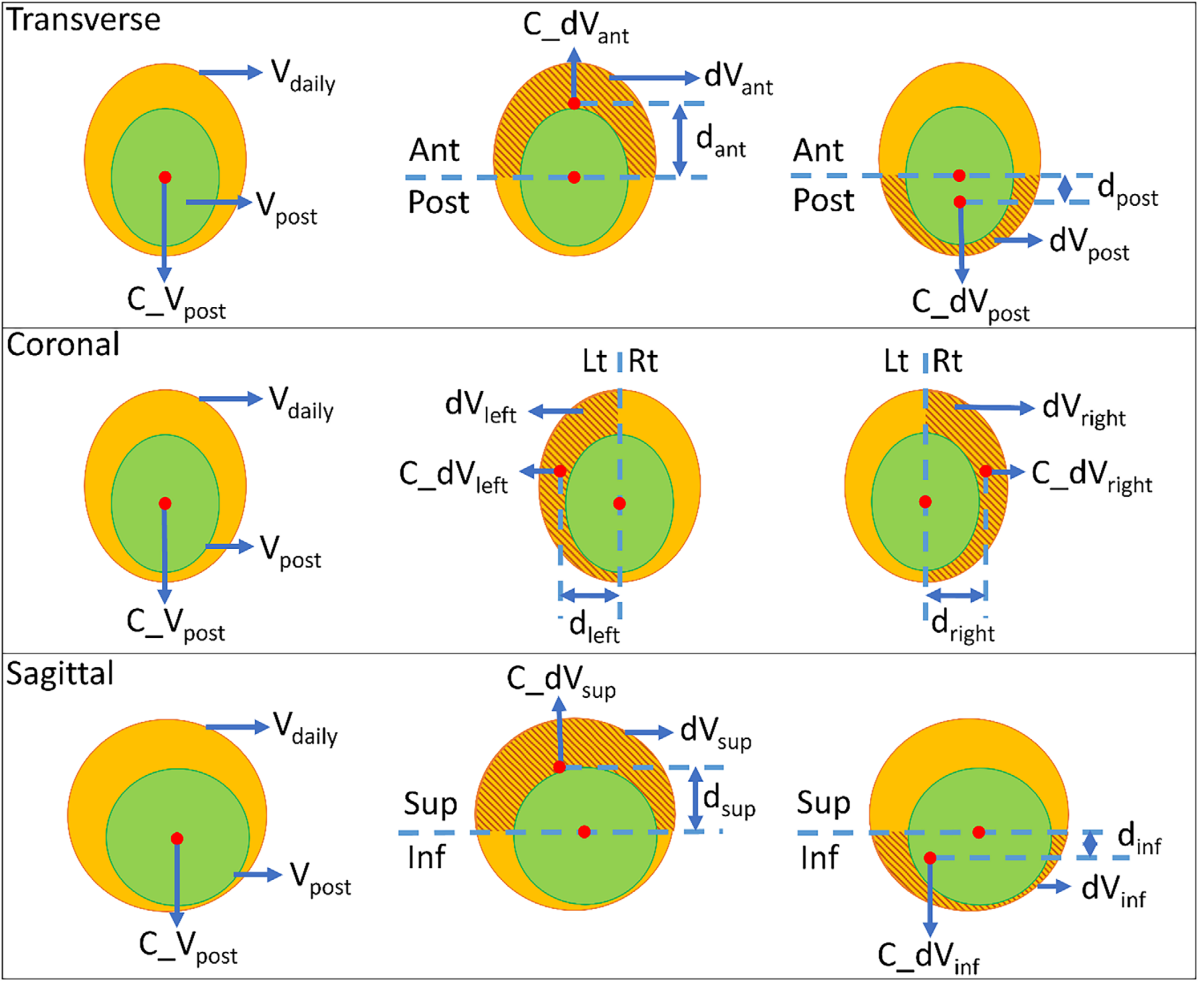
Illustration of parameters defined in Equation (1) for calculating normalized directional distance. The orange color‐wash is the (V_daily_). The green color‐wash is the (V_post_). The shaded area denotes the directional residual volume (dV_xx_), defined as the difference between V_daily_ and V_post_ in the xx (e.g. sup) direction. C_V_post_ is the centroid of V_post_, and C_dV_xx_ is the centroid of dV_xx_. d_xx_ is the distance between C_dV_xx_ and C_V_post_ along the xx (e.g. sup) direction. (V_daily_), daily bladder volume; (V_post_), post‐void bladder volume.

With the planning bladder volume V_post_ as the benchmark, we proposed a method to assess the directional changes in V_daily_ for each patient. As illustrated in Figure 1, we subtracted V_post_ from V_daily_ and split the subtracted volume into anterior/posterior, left/right, or superior/inferior segments based on the centroid of V_post_. To translate the subtracted volume into a margin recipe applicable to the V_post_, we defined a normalized distance termed $d_{dir}^{n}$, in all six directions Equation (1). This was used to create a comprehensive profile of normalized directional distance.

(1) $$\;d_{dir}^{n}=d_{dir}\;\times \frac{dV_{dir}}{V_{post}}$$

$d_{dir}^{n}$: normalized directional distance in millimeter.

$d_{dir}$: distance between centroid of directional residual volume and the centroid of post‐void bladder volume, in the corresponding direction, in millimeter.

$dV_{dir}$: directional residual volume, the portion of (V_daily_) that resides outside of the post‐void bladder in certain direction, in cc.

$V_{post}$: post‐void bladder volume in cc.

#### 2.2.2 Adaptive margins based on daily kV‐CBCTs from the first five fractions

After the first five fractions of treatments, we proposed a patient‐specific adaptive margin recipe for subsequent fractions. This method incorporated the changes in the on‐treatment bladder of individual patients during these initial five fractions. This process involved combining two simulation CT scans and five daily KV‐CBCTs. For each patient, we calculated the mean and standard deviation (SD) of the normalized directional distance, $d_{dir}^{n}$ according to Equation (1), using data from the five KV‐CBCTs. We used the Pearson correlation coefficient to assess the correlation between the $d_{dir}^{n}$ and the directional residual volume $dV_{\mathrm{dir}}$. We tested four potential adaptive margins based on the $d_{dir}^{n}$ of each patient, as displayed in Table 1. We evaluated the PTVs created from these four methods against the V_daily_ from the sixth to the tenth fractions and the final five fractions of KV‐CBCTs. We calculated the ∆V (%) using the V_daily_ from the sixth to the tenth fractions in relation to the four proposed PTVs. This calculation helped us determine an optimal patient‐specific margin recipe based on the size of the residual volumes. Finally, we validated the proposed patient‐specific adaptive margin recipe using the KV‐CBCTs from the final five fractions.

**TABLE 1:** Patient‐specific adaptive margin recipes based on daily image analysis.

Method	Equation
M1	$margin=\bar{d_{dir}^{n}}+2\times SD$
M2	$\;D_{dir}=\bar{d_{dir}^{n}}+2\times SD$ $margin=\left\{\begin{array}{c} 3,\;if\;D_{dir}<3\\ D_{dir},\;if\;D_{dir}\geq 3 \end{array}\right.$
M3	$margin=\bar{d_{dir}^{n}}+2\times SD+2$
M4	$margin=\bar{d_{dir}^{n}}+3\times SD+5$

Abbreviations: $\bar{d_{dir}^{n}}$: average normalized directional distance (mm) from the first five fractions; SD: standard deviation of normalized directional distance.

### 2.3 Adaptive plan optimization

Once an optimal patient‐specific anisotropic margin recipe has been selected from the four methods proposed in Table 1, an offline adaptive plan was created using the selected margin recipe on the post‐void simulation CT. The planning prescription was 64 Gy in 32 fractions. Sixteen adaptive plans were created using scripts developed in‐house in the Pinnacle Treatment Planning System (version 16.2.1, Philips Medical Systems, Inc. Fitchburg, WI). The goal was to meet the planning objectives outlined in our locally established planning scorecard for bladder plans, as shown in Table 2. The primary objective was to achieve a uniform prescription dose received by the PTV (95% of the PTV covered by prescription dose, with maximum point dose < 105% of prescription dose, or < 107% if 105% was not achievable). We have developed an in‐house, unsupervised auto‐planning script for this study, incorporating a two‐step optimization process. The first step in this unsupervised auto‐planning process directly used the auto‐planning module within the Pinnacle system to generate a base plan. The second step of optimization included the iterative addition of plan optimization objectives to the optimizer, with the aim of reducing the dose to critical organs until PTV coverage was compromised. This planning process eliminates the need for human intervention after the iso‐center placement and setting of the treatment beams. Once the adaptive target volumes were approved by the attending physician, the adaptive planning process started without further human intervention. This process generated multiple plans, further reducing the dose to OARs beyond the clinical goals listed in the scorecard (Table 2). After reviewing the multiple plans created by the adaptive planning process, a planner selected the most balanced adaptive plan.

**TABLE 2:** The primary and secondary clinical goals.

ROI	Primary goal	Secondary goal ^a^
CTV	V 6400 cGy = 100% ^b^	V 6400 cGy ≥ 99%
PTV	V 6400 cGy ≥ 95%	–
PTV	V 6720 cGy ≤ 0.03 cc	V 6912 cGy ≤ 0.03 cc
Rectum	V 6000 cGy ≤ 15%	–
Rectum	V 4000 cGy ≤ 40%	–
LG_BOWEL	V 6720 cGy ≤ 0.03 cc	–
LG_BOWEL	V 6500 cGy ≤ 10 cc	–
LG_BOWEL	V 6000 cGy ≤ 10%	–
LG_BOWEL	V 4000 cGy ≤ 50%	–
SM_BOWEL	V 6720 cGy ≤ 0.03 cc	–
SM_BOWEL	V 4500 cGy ≤ 50 cc	–
SM_BOWEL	V 5000 cGy ≤ 1%	V 6720 cGy ≤ 1%
FEMUR_R	V 5000 cGy ≤ 1%	–
FEMUR_R	V 3000 cGy ≤ 10%	–
FEMUR_L	V 5000 cGy ≤ 1%	–
FEMUR_L	V 3000 cGy ≤ 10%	–

^a^ Secondary goal applies if the primary goal cannot be achieved.

^b^ V x cGy represents either the percentage volume (%) or the absolute volume (cc) of the ROI receiving an x cGy dose.

Abbreviations: CTV, clinical target volume; PTV, planning target volume.

To evaluate the effectiveness of the proposed patient‐specific anisotropic adaptive margin recipe, we compared our adaptive plans with plans generated using conventional anisotropic margins. The conventional margins, as recommended by Khalifa et al., were 1.5 cm superiorly and anteriorly, and 1 cm in other directions with daily image‐guided radiation therapy (IGRT).^1^

### 2.4 Plan evaluation

We compared adaptive plans with conventional plans to evaluate differences in doses to OARs and average prescription dose coverage to V_daily_. V_daily_ contours were extracted from the kV‐CBCTs acquired between fractions six and ten, and the final five fractions, and transferred to their respective plans. Student's *t*‐tests were used for statistic analysis.

## 3 RESULT

### 3.1 (V_daily_) variation

**FIGURE 2:**
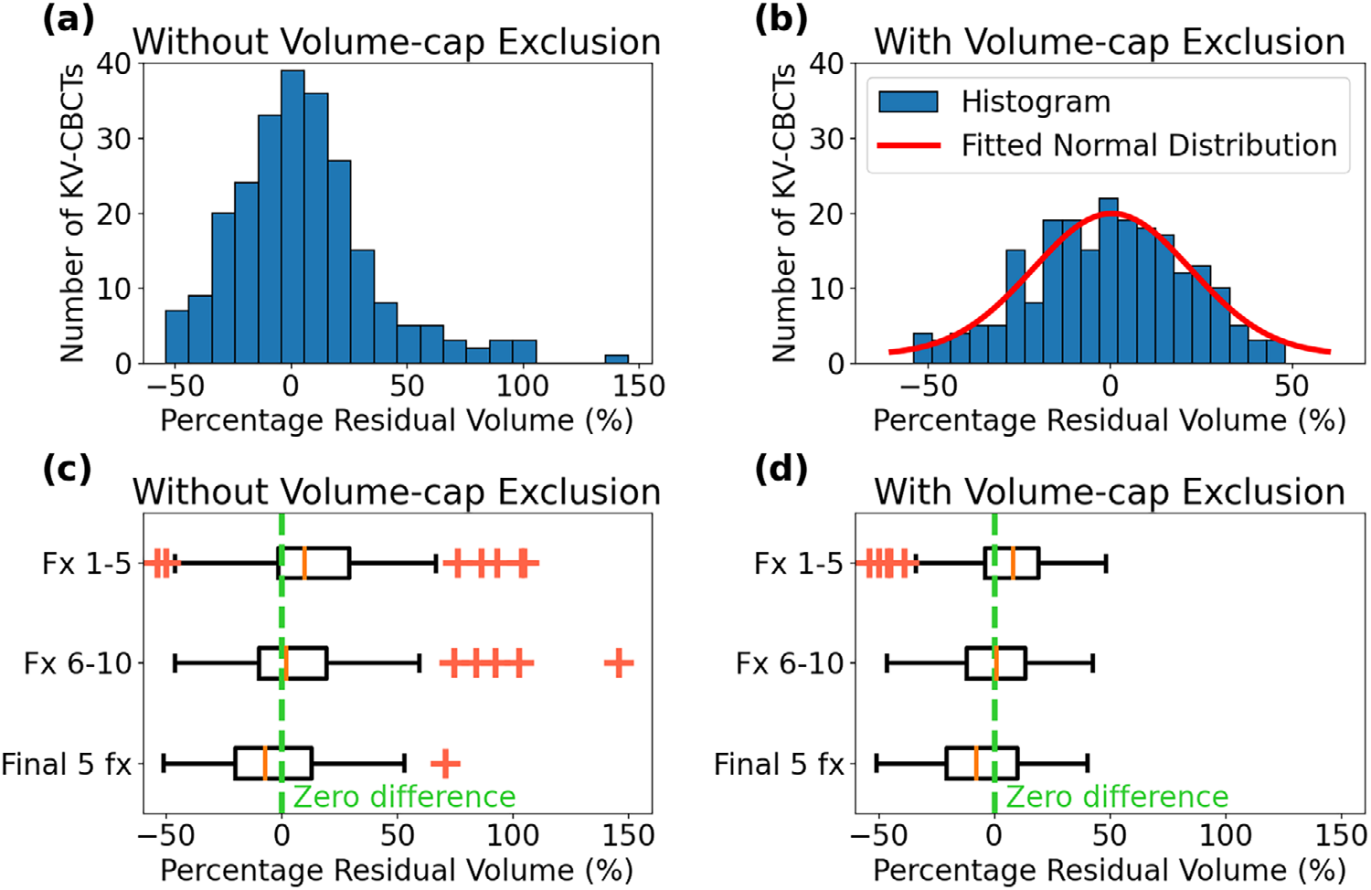
Percentage difference between (V_daily_) and (V_post_). (a) Distribution of the number of V_daily_ by percentage bladder volume differences without the volume‐cap exclusion. (b) Distribution of the number of V_daily_ with the volume‐cap exclusion. (c) Percentage volume differences for three fraction groups without exclusion. (d) Percentage volume differences for three fraction groups with exclusion. (V_daily_), daily bladder volume; (V_post_), post‐void bladder volume.

Out of the total of 240 daily kV‐CBCTs, 132 (55.0%) showed daily bladder volumes (V_daily_) exceeding their respective post‐void volumes (V_post_). The mean V_daily_ was observed to be 6.1% ± 30.7% greater than the V_post_. The distribution of the residual bladder volume, ∆V(%), was positively skewed, indicating the presence of outliers where V_daily_ exceeded V_post_ (Figure 2a). Upon implementing an exclusion criterion wherein V_daily_ > 150% of the respective V_post_, 21 kV‐CBCTs (8.8% of the total count) would be omitted from the analysis. Following this exclusion, half of the V_daily_ (111 kV‐CBCTs, 50.7%) were found to still greater than their corresponding V_post_. However, the mean residual volume decreased to −0.6% ± 21.4%, implying that the V_daily_ was more similar to the V_post_. When the exclusion criterion was applied, the distribution of the residual volume demonstrated a pattern closer to a normal distribution, as depicted in Figure 2b.

In the analysis of the 80 V_daily_ from the first five fractions’ KV‐CBCTs, the DSC between each V_daily_ and its corresponding V_post_, was found to range from 0.59 to 0.95, with a mean of 0.78 and an SD of 0.09. When the V_daily_ samples that were excluded from the analysis (12 in total) were considered separately, they exhibited a lower DSC of 0.69 ± 0.06. In contrast, the DSC of the remaining 68 V_daily_ samples was higher (0.80 ± 0.08). Furthermore, a strong correlation was observed between the DSC and the daily residual volume, as the correlation coefficient r(78) = 0.68, and a *p*‐value *p* < 0.005.

During the course of radiation treatments, as illustrated in Figure 2c,d, the bladder volumes decreased with and without the application of exclusion criteria. In Table 3, we have summarized the number of outliers of V_daily_, the number of fractions where V_daily_ exceeded V_post_, and the volume difference expressed as a percentage of V_post_, grouped by weeks. The number of bladder volume outliers decreased from 15% in the first five fractions of treatments to 2.5% in the final five fractions. Similarly, the number of fractions where V_daily_ was larger than V_post_ decreased from 67.6% in the first five fractions to 44.9% in the final five fractions. The percentage of volume difference between V_daily_ and V_post_ shifted from positive to negative. These data confirmed that bladder volume consistently decreased over the course of treatment.

**TABLE 3:** Comparisons between V_daily_ and V_post_ by three fraction groups.

	First five fractions	Sixth to tenth fractions	The final five fractions
Number of outliers^a^	12 (15%)^b^	7 (8.8%)	2 (2.5%)
Number of fractions with bladder volume > V_post_	46 (67.6%)	37 (50.7%)	35 (44.9%)
Percentage of volume difference^c^	5.1% ± 22.3%	−0.3% ± 19.3%	−5.9% ± 21.2%

^a^ An outlier is defined as V_daily_ exceeding 150% of V_post_.

^b^ The number in parenthesis represents the percentage to the total number of kV‐CBCTs in respective group.

^c^ Percentage of volume difference is defined as 100% × (V_daily_—V_post_)/ V_post_, represented as mean ± standard deviation.

Abbreviations: (V_daily_), daily bladder volume; (V_post_), post‐void bladder volume.

### 3.2 Patient‐specific adaptive margins

We have calculated the normalized directional distance $d_{dir}^{n}$ from the first five kV‐CBCTs for each patient. The correlations between $d_{dir}^{n}$ and the residual volume, ∆V(%), are presented in Table 4. In the anterior and superior direction, a strong correlation was observed between $d_{dir}^{n}$ and the residual volume, exhibiting statistical significance regardless of the application of the exclusion criterion. However, the correlation between both the normalized left distance and the residual volume, and the normalized right distance and the residual volume became less strong following the application of exclusion criterion. The normalized posterior and inferior distances demonstrated minimal correlation with the residual volume

**TABLE 4:** Correlation between normalized directional distance and residual volume.

	Anterior		Posterior		Left		Right		Superior		Inferior	
	w/o ^d^	w/ ^e^	w/o	w/	w/o	w/	w/o	w/	w/o	w/	w/o	w/
r^a^	0.86	0.83	0.56	0.49	0.87	0.79	0.89	0.81	0.91	0.86	0.50	0.40
R^2^ ^b^	0.74	0.68	0.31	0.24	0.75	0.62	0.79	0.65	0.83	0.74	0.24	0.16
P^c^	<0.01	<0.01	0.02	>0.05	<0.01	<0.01	<0.01	<0.01	<0.01	<0.01	>0.05	>0.05

^a^ r: correlation coefficient;.

^b^ R^2^: R^2^ of linear regression;.

^c^ The significance is at the *p* = 0.05 level.

^d^ w/o represents the group without applying the exclusion criteria;.

^e^ w/ represents the group with the application of exclusion criteria.

**FIGURE 3:**
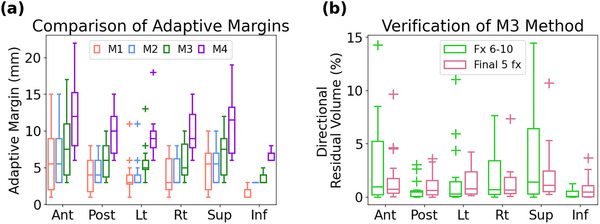
Adaptive margins and residual volume in six orthogonal directions. (a) Distribution of adaptive margins from the first five fractions based on the four margin calculation methods (M1‐M4) defined in Table 1. (b) Distribution of directional residual volume in the sixth to tenth fractions and the final five fractions using the M3 method.

The four proposed anisotropic expansion methods (M1‐M4) listed in Table 1 were evaluated by examining the distribution of the anisotropic margins in each direction, as shown in Figure 3a. The evaluation process utilized kV‐CBCTs acquired from the sixth to tenth treatment fractions to determine the optimal adaptive margin recipe capable of achieving less than 5% of daily residual volume. Out of the 80 kV‐CBCTs, seven were excluded by the application of the 150% V_post_ volume cap criteria. We registered the daily kV‐CBCT with the corresponding post‐void simulation CT, with alignment focused on the bladder neck. We then transferred the daily bladder contours from kV‐CBCTs to the post‐void CT. The four proposed patient‐specific adaptive margin recipes resulted in average and SD of the percentage residual volume of daily bladder contour residing outside of the PTV as follows: 7.2% ± 6.4%, 5.9% ± 5.4%, 3.7% ± 4.3%, and 1.7% ± 2.6%, respectively (Figure 3b). Based on the goal of achieving less than 5% of daily residual volume, the M3 PTV expansion method proved to be the most optimal. The mean anisotropic margins and their corresponding SDs ranked from highest to lowest, were 7.9 ± 4.1 mm in the anterior direction, 6.9 ± 2.9 mm in the superior direction, 6.0 ± 2.3 mm in the right direction, 5.8 ± 2.1 mm in the posterior direction, 5.7 ± 2.3 mm in the left direction, and 3.7 ± 0.6 mm in the inferior direction. The directional residual volume was largest superiorly with a mean of 3.7% ± 4.2% and anteriorly with a mean of 3.1% ± 3.9%, while it was smallest inferiorly with a mean of 0.3% ± 0.4% (Figure 3b).

In order to further validate the efficacy of the M3 recipe, we transferred the daily bladder contours from the final five treatment fractions to the post‐void simulation CT, with the application of the predetermined exclusion criterion. The mean residual volume, expressed as a percentage of V_post_, was 2.7% with a standard deviation of 2.3%. The measurements of directional residual volume for the daily bladder during the final five fractions were similar to those observed in the sixth to tenth fractions. The largest residual volume was observed in the anterior and superior directions, with mean values and SDs of 1.8% ± 2.5% and 2.2% ± 2.7% respectively. Conversely, the smallest directional residual volume was noted in the inferior direction, with a mean of 0.8% and a SD of 1.0%.

### 3.3 Dosimetric evaluation of adaptive plans

**FIGURE 4:**
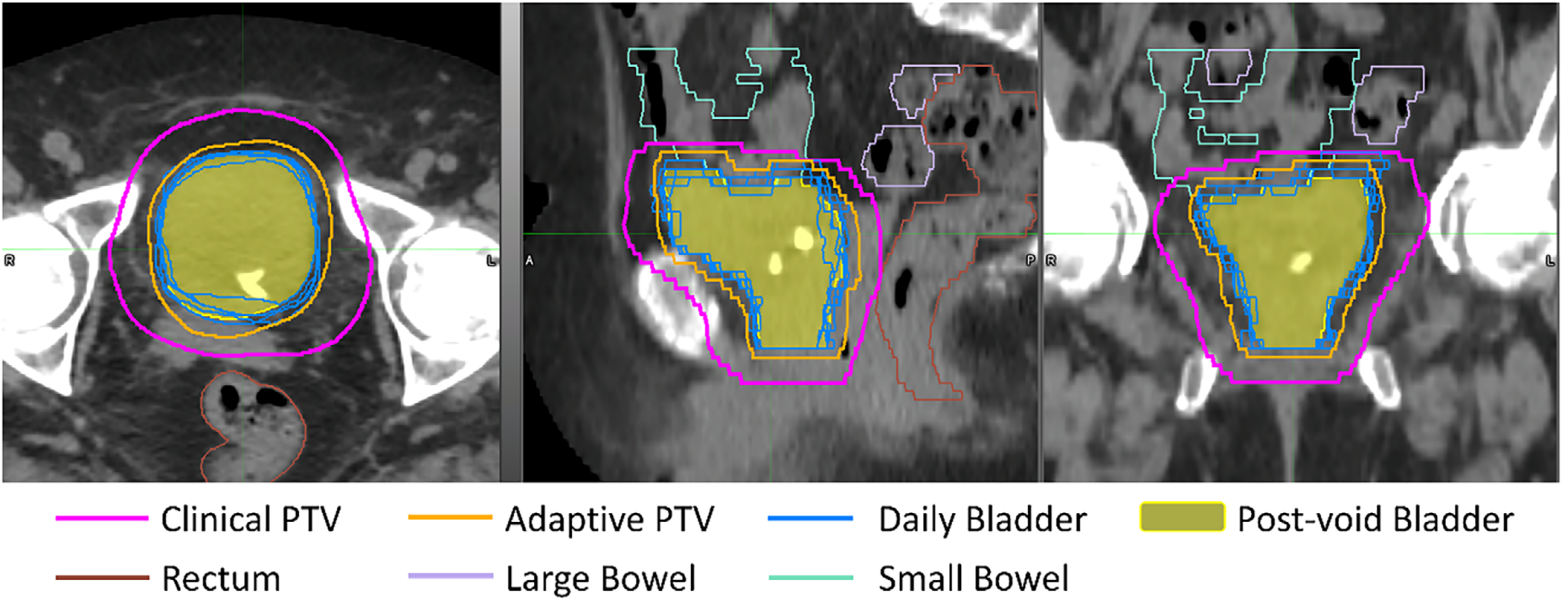
Comparison of the clinical PTV (purple) and the adaptive PTV (orange) on post‐void planning CT. The yellow color‐wash represents the (V_post_), the blue contours are the (V_daily_) from the first five fractions. Critical OARs include the rectum (brown), large bowel(violet), and small bowl (teal). OAR, organ at risks; PTV, planning target volume; (V_daily_), daily bladder volume; (V_post_), post‐void bladder volume.

Figure 4 illustrates an example patient's post‐void bladder contour, daily bladder contours from the first five fractions, clinical PTV, and an adaptive PTV generated using the adaptive margin (M3). The adaptive PTV closely encompasses the daily bladder variation with minimum daily residual volume and minimum normal tissue involvement, while the clinical PTV encapsulates all the daily variation but with much greater normal tissue involvement. An in‐house, unsupervised auto‐planning script was used to create sixteen adaptive plans using M3 adaptive margins. The dose constraints to OARs are listed in Table 2. All sixteen adaptive plans achieved sufficient dose coverage to the PTV, and 15 met the uniformity goal. However, one patient's plan did not meet one of the large bowel constraints (V_65Gy_ < 10 cc) due to a volume overlap with the PTV of 14.3 cc. Two patients’ plans exceeded the other large bowl constraint (V_60Gy_ < 10%). For one patient, this was due to the large bowel overlapping with the PTV by 12.6%. All other constraints were successfully met for all adaptive plans.

The same auto‐planning script was used to generate sixteen conventional plans using the conventional margin. All conventional plans achieved adequate PTV dose coverage, and 15 met the dose uniformity goal. However, these conventional plans demonstrated significant OAR dose violations: four plans exceeded the large bowel V_65Gy_ < 10 cc constrain, ten exceeded the large bowel V_60Gy_ < 10% constraint, one exceeded the large bowel V_40Gy_ < 50% constraint, and ten exceeded the small bowel V_45Gy_ < 50 cc constraint. These failures were primarily due to extensive overlaps between the bowel structures and the PTV.

**FIGURE 5:**
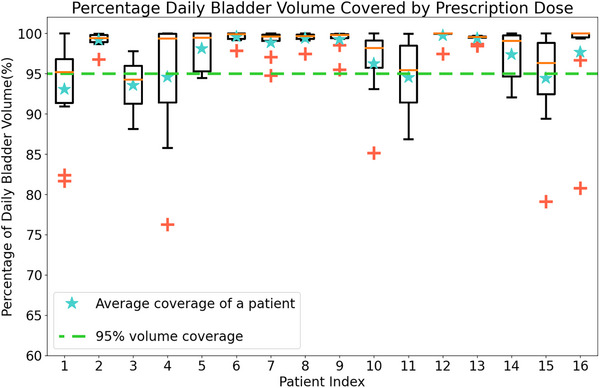
Boxplot of (V_daily_) coverage by the prescription dose using M3 adaptive margins for the sixth to tenth and final five fractions of treatment. The orange line within each box represents the median value. Red “+” symbols are outliers beyond 1.5 times the interquartile range (IQR). Whiskers extend to the minimum and maximum values excluding outliers. The green dashed line is a baseline of 95% volume coverage. The blue stars (★) represent the average value. (V_daily_), daily bladder volume.

Figure 5 presents the coverage of the prescribed dose for daily bladder contours for adaptive plans, using data from the sixth to tenth fractions and the final five fractions. Without applying the exclusion criterion, the median coverage to the daily bladder was 99.1% (with an interquartile range of 95.3%–99.9%, a minimum of 65.5%, and an average of 96.1%). However, with the application of the exclusion criterion (which excluded 9 daily kV‐CBCTs), the median coverage increased slightly to 99.3% (with an interquartile range of 96.1%–99.9%, a minimum of 76.3%, and an average of 97.2%). Out of the 151 daily kV‐CBCTs, 81% achieved more than 95% of the (V_daily_) receiving the prescribed dose.

For conventional plans, without the exclusion criterion, the median coverage to the daily bladder was 99.9% (interquartile range: 98.4%–100%, minimum: 83.2%, average: 98.5%). Applying the exclusion criterion had a negligible impact, maintaining the median coverage at 99.9% (interquartile range: 98.8%–100%, minimum: 83.2%, average: 98.7%). In this case, 92.7% of the 151 daily kV‐CBCTs achieved greater than 95% of the V_daily_ receiving the prescribed dose.

For the adaptive plans, after excluding the 9 daily kV‐CBCTs that did not meet the exclusion criteria, a negative correlation was observed between the coverage of the prescribed dose on the remaining V_daily_ and the percentage of volume difference between V_daily_ and V_post_, with r(158) = 0.7 and *p* < 0.01. However, these two parameters did not display a similar correlation in the excluded group, with r(7) = 0.55 and *p* > 0.1. In the excluded group, the median prescribed dose coverage on the daily bladder was 73.33%, with an interquartile range of 70.8% to 84.9% and a minimum value of 65.5%.

### 3.4 Plan comparison between adaptive plans and conventional plans

Conventional PTVs were, on average, 68.1% (135.3 cc ± 46.6 cc) larger than adaptive PTVs (*p* < 0.05). Conventional plans also resulted in significantly higher OAR doses (*p* < 0.05) compared to adaptive plans. Specifically, rectum dose‐volumes increased by an average of 2.8% at V_60_ _Gy_ and 10.3% at V_40Gy_. Large bowel dose‐volumes increased by an average of 10.1% at V_60_ _Gy_ and 15.0% at V_40Gy_. Small bowel V_45_ _Gy_ and V_50_ _Gy_ increased by an average of 28.6 cc and 7.4%, respectively. Additionally, the average dose coverage to the daily bladder was 1.5% higher in conventional plans.

## 4 DISCUSSION

This study included sixteen patients with MIBC who underwent whole‐bladder radiation therapy. Daily kV‐CBCTs were acquired throughout the treatment course, with the bladder being contoured on each image. The (V_daily_) was found to be, on average, 6.1% (± 30.7%) larger than the simulated (V_post_). A patient‐specific margin recipe was proposed based on the bladder volume variations observed from the kV‐CBCTs on the first five fractions of treatment. This was validated using the kV‐CBCTs from the sixth to tenth fractions and the final five fractions. The proposed patient‐specific margin recipe resulted in an average of 6 (± 1.2) mm, which was smaller than the conventional PTV margins (15 to 20 mm), while still able to account for the daily bladder variations. The adaptive plan that utilized the patient‐specific margin provided adequate dose coverage to the target and effectively protect the surrounding normal tissue, meeting clinically acceptable goals.

Mangar et al. suggested that decreasing the isotropic margin to 10 mm would not significantly impact dose coverage, provided a (V_daily_) cap of 150% of the simulated bladder volume is maintained.^3^ In our study, we discovered that the bladder volume variation can be characterized as a normal distribution by enforcing a volume cap of 150% of the (V_post_). The daily bladder variation would be random with the application of the volume cap, but the random variation can be characterized within a predictable range unique to each individual patient. Several studies have explored the trend of bladder volume during radiotherapy, but the results have been inconsistent. While Manga et al. found no consistent trend in (V_daily_), other studies have suggested a decrease in (V_daily_) towards the end of the radiotherapy course.^1^ In our retrospective study, we observed a decline in the average (V_daily_) towards the end of the treatment. During the first five fractions of treatment, V_daily_ was 5% greater than V_post_. V_daily_ was similar to V_post_ from the sixth to tenth fractions, and during the final five fractions, V_daily_ was 5% less than V_post_. This indicates a total reduction in (V_daily_) of around 15%. The inconsistent findings could be attributed to differences in the empty‐bladder treatment protocols, which can affect bladder filling and the volume throughout the course of treatment. It is crucial to adhere to a consistent protocol that treats patients immediately after bladder voiding. The observed volume trends also highlight the need for patient‐specific adaptive margins as patients progress through the treatment course.

As IGRT techniques become increasingly accessible to radiation oncology departments, several studies have been conducted to characterize the inter‐fractional bladder variation.^1^, ^3^, ^5^, ^9^, ^12^ It has been observed that daily changes in bladder shape and volume predominantly occur in the superior and anterior directions. As a result, recommendations for anisotropic margins have been proposed, with a larger margin in the superior and anterior directions and a smaller margin in other directions. Both population‐based and ITV‐based anisotropic margins have been designed with the aim of reducing the volume of normal tissue exposed to the full prescription dose, without compromising the efficacy of the radiotherapy.^2^, ^9^, ^14^, ^19^, ^20^, ^21^, ^22^, ^23^, ^24^

The population‐based anisotropic margin method utilizes CBCT data from a collection of patients previously treated to develop margins similar to the van Herk margin recipe. This method takes into account both setup error and organ motion.^25^ The resulting anisotropic margins are typically largest in the superior and anterior directions, approximately 15 mm, and less than 10 mm in other directions. Once these anisotropic margins are calculated, they are applied uniformly to all future patients. However, this approach has limitations as it requires a retrospective analysis of CBCT data from a large patient cohort and may not consider individual patient's clinical conditions, such as the physiological functioning of the bladder. The van Herk recipe might not be able to adapt to variations in the bladder beyond organ motion, considering that it was originally designed for a rigid target.^26^ Moreover, using the same margin for all patients may not be the most optimal strategy, as it does not account for each patient's unique circumstances.^27^

In contrast to population‐based margins, ITV‐based anisotropic margins offer a personalized approach that takes into account each patient's individual day‐to‐day bladder variations. To create the ITV, the daily bladder contours from the first few fractions’ CBCTs were combined, with additional expansions incorporated to account for potential residual variations.^14^, ^15^, ^19^, ^24^ This method reduces the volume of irradiated normal tissue while maintaining equivalent dose coverage to the bladder as compared to the population‐based margin.^19^ However, bladder variation, unlike respiratory motion, is neither rhythmic nor regular. Consequently, an ITV that only includes bladder variations from the first few fractions might result in overfitting.

Several studies have explored various margin recipes for whole bladder irradiation to balance margin size and target coverage. Murthy et al. suggested that using anisotropic PTV margins that cover 95% or 90% of CTV could effectively balance CTV coverage, normal tissue toxicity, and resource utilization.^17^ Vestergarrd et al. combined population‐based margins (5–20 mm anisotropic expansion) with ITV‐based margins (union of daily bladder volumes with an additional 8 mm expansion) to achieve high (V_daily_) coverage in 97% of all fractions.^22^ Pos et al. employed ITV‐based margins (combining bladder volume from planning CT and 5 daily CTs with a 10 mm expansion) and observed a 5% daily bladder outside the PTV.^23^ Gronborg et al. investigated population‐based margins (5–14 mm anisotropic expansion) and reported coverage rates between 98.1% and 99.9%.^28^ Foroudi et al. compared three ITV‐based margins, each with a minimum of 15 mm expansion, and observed CTV volume coverage by the 95% prescribed dose ranging from 63.91% to 100%.^15^

In this study, we proposed a novel patient‐specific anisotropic margin, tailored to the individual patient's bladder anatomy and variability. This margin, averaging 6.0 mm (± 2.9 mm), was calculated by comparing post‐void simulation CT and daily bladder measurements from the first five fractions. By adapting treatment plans using this margin recipe, we achieved a high (V_daily_) coverage of 95% or more by full prescription dose in 81% of fractions, with an average coverage volume rate of 97.2%. Our proposed adaptive margin, smaller than the recommended conventional margins (1.0–1.5 cm), significantly reduced irradiated normal tissue volume while maintaining an average of over 95% coverage of (V_daily_).

To create this patient‐specific margin, we employed a straightforward yet effective method. We calculated the normalized directional distance by multiplying the directional residual volume and its corresponding directional centroid distance. This approach ensures that the directional margin increases only when both the residual volume and centroid distance in a specific direction are large, preventing excessive expansion. By computing margins in each direction separately, we accounted for the bladder wall's asymmetric elasticity, which is influenced by its inherent anatomy and potential disease progression. As the bladder volume decreases throughout the course of radiotherapy, clinicians can adjust the target volume as needed, using the same algorithm midway through the treatment course by adopting different sets of CBCT images acquired daily. Alternatively, a plan‐of‐the‐day approach can be employed, selecting either the adaptive or conventional plan based on daily kV‐CBCT evaluation of bladder volume.

While traditional PTV expansion methods focus on six standard directions (superior, inferior, anterior, posterior, left, and right), we adopted this approach for our patient‐specific adaptive margins to maintain simplicity and effectiveness. These margins were calculated and applied to the whole bladder using either MIM or Pinnacle software, both of which are restricted to expansions in these six directions. However, we recognized the limitations of this approach in fully capturing the unique anisotropic profile of individual patients's bladder variation. For example, the post‐void bladder appeared concave at the superior aspect. As the bladder filled, the concave portion would fill and become convex. Expanding in the superior direction alone would not adequately address this change, as it would simply extend the original concave surface superiorly, rather than making it convex. To overcome this limitation, we could expand the target volume in additional directions using alternative image processing software and directly export the expanded PTV into treatment planning software. This approach would enable us to investigate the relationship between the extent of disease invasion and the elasticity of the bladder wall.

Online adaptive radiotherapy has grown increasingly popular in recent years due to its ability to adapt treatment plans based on daily images. This potentially reduces margins, sparing the surrounding normal tissues. However, the collective time required for contouring, automated adaptive planning, and plan quality assurance is not trivial.^19^, ^20^, ^29^, ^30^ Åström noted that a median of 13.9 min of additional time was required for online adaptive radiation treatment for whole bladder irradiation on a CBCT‐based Varian Ethos system. Hunt reported that a median of 39 min of extra time was needed on the MRI‐based Elekta Unity system. In addition to contouring the bladder on daily imaging, anisotropic margins were incorporated into these online adaptive reference plans to accommodate intra‐fractional bladder volume variation during the extended period between imaging and treatment delivery. The sizes of the margins ranged from 5–15  mm, comparable to the patient‐specific adaptive margin we designed in this study. An offline adaptive plan can minimize the delay between the daily image acquisition and the start of treatment, ensuring that the daily image accurately reflects the patient's anatomy during treatment. Our method of using each individual's bladder historical profile to predict the range of variation is an offline adaptive approach, which ensures the validity of the daily CBCT. The tailored patient‐specific adaptive margin further reduces normal tissue exposure while maintaining target coverage by prescribed dose.

## 5 CONCLUSION

By enforcing a volume cap of 150% of (V_post_), the (V_daily_) variation can be characterized as a normal distribution. Our patient‐specific margin recipe for whole bladder irradiation can capture this characteristic while being smaller compared to conventional margins, and it can ensure adequate PTV coverage and optimized normal tissue sparing.

## AUTHOR CONTRIBUTIONS

**Zhexuan Zhang**: Conception and design; Data collection; Data analysis and interpretation; Manuscript writing. **Chieh‐Wen Liu**: Data analysis and interpretation; Manuscript writing. **Jeremy D. Donaghue**: Data collection; Final approval of the manuscript. **Eric J. Murray**: Data collection; Final approval of manuscript. **Omar Mian**: Conception and design; Final approval of manuscript. **Ping Xia**: Conception and design; Manuscript writing; Final approval of manuscript.

## CONFLICT OF INTEREST STATEMENT

O.M. receives research funding from Varian and Gilead to support the adaptive bladder radiotherapy trial. Other authors declare no conflict of interest relevant to this study.
